# Alzheimer and Purinergic Signaling: Just a Matter of Inflammation?

**DOI:** 10.3390/cells10051267

**Published:** 2021-05-20

**Authors:** Stefania Merighi, Tino Emanuele Poloni, Anna Terrazzan, Eva Moretti, Stefania Gessi, Davide Ferrari

**Affiliations:** 1 Department of Translational Medicine and for Romagna, University of Ferrara, 44100 Ferrara, Italy; stefania.merighi@unife.it (S.M.); anna.terrazzan@edu.unife.it (A.T.); eva.moretti@edu.unife.it (E.M.); 2 Department of Neurology and Neuropathology, Golgi-Cenci Foundation & ASP Golgi-Redaelli, Abbiategrasso, 20081 Milan, Italy; e.poloni@golgicenci.it; 3 Department of Life Science and Biotechnology, University of Ferrara, 44100 Ferrara, Italy

**Keywords:** Alzheimer’s disease, neuroinflammation, P1 receptors, P2 receptors

## Abstract

Alzheimer’s disease (AD) is a widespread neurodegenerative pathology responsible for about 70% of all cases of dementia. Adenosine is an endogenous nucleoside that affects neurodegeneration by activating four membrane G protein-coupled receptor subtypes, namely P1 receptors. One of them, the A_2A_ subtype, is particularly expressed in the brain at the striatal and hippocampal levels and appears as the most promising target to counteract neurological damage and adenosine-dependent neuroinflammation. Extracellular nucleotides (ATP, ADP, UTP, UDP, etc.) are also released from the cell or are synthesized extracellularly. They activate P2X and P2Y membrane receptors, eliciting a variety of physiological but also pathological responses. Among the latter, the chronic inflammation underlying AD is mainly caused by the P2X7 receptor subtype. In this review we offer an overview of the scientific evidence linking P1 and P2 mediated purinergic signaling to AD development. We will also discuss potential strategies to exploit this knowledge for drug development.

## 1. A Brief Update on Alzheimer’s Disease (AD)

The new classification proposed by the DSM-5 uses the term neurocognitive disorder (NCD), emphasizing that the origin of mental deficits lies in a dysfunction affecting neuronal networks. The very first stages of NCD (mild-NCD, i.e., mild cognitive impairment (MCI)) are characterized by preserved functional abilities and the capacity to be independent in daily activities. If, on the other hand, the disorder determines a functional decrement, it is defined as a major-NCD (or dementia). Further, the identification of the underlying pathology is needed for the etiological classification of the NCD [1,2]. The pathologic hallmark of NCDs that are due to AD is a double proteinopathy in which neurodegeneration is related to the deposition of amyloid-beta (Aβ) and phosphorylated TAU protein (pTAU). AD is the most frequent age-related degenerative NCD that causes an impairment of multiple cognitive domains, gradually involving memory, complex attention, executive functions, language, and visual-perceptual functions. Consequently, from the beginning of the disease’s course, deep changes progressively develop in personality and behavior. Conversely, due to the late involvement of the basal ganglia, motor dysfunction (i.e., parkinsonism) typically appears during the later stages of the disease. AD’s syndromic evolution follows the topography of pTAU pathology from the allocortex (entorhinal cortex and hippocampus) to the neocortex, and then to basal ganglia in the terminal stages [3,4]. Although AD is a typical age-related pathology, the disease takes its first steps from the age of 40 onwards, with an accumulation of amyloid in the neocortex. However, amyloid deposition is very commonly found in physiological aging, thus, it is not enough to cause AD. The fundamental question of what triggers neurodegeneration remains without a certain answer. According to the amyloidogenic theory, neurodegeneration occurs in the presence of an excessive amount of Aβ, through the formation of Aβ toxic oligomers. Indeed, an Aβ load induces neurodegeneration not *per se* but through soluble oligomers that disturb cellular functioning through a harmful interaction with the membrane of neurons, especially at the synaptic level. One of the most investigated mechanisms of damage to the neuronal membrane is the possible aggregation of some oligomers (e.g., β_25–35_) to form anomalous ion channels (increasing the entry of Ca^2+^) or even real “pores”, which can allow the chaotic entry of ions and harmful substances into the neuron [5,6]. All of these detrimental perturbations may induce synaptic dysfunction. The mechanisms by which oligomers cause neuronal damage are many and still little known. Instead, the final consequences of brain amyloid excess are partially known and can be summarized as follows: 1) a decrease in synaptic transmission with a dysfunction in the long-term potentiation and, consequently, a deficit in memory and other cognitive domains; 2) a decrease in the blood flow in brain capillaries; and 3) an increase in phosphorylation of the AD-relevant epitopes of the TAU protein [7,8]. The over-activation of neuronal kinases, resulting in unbalanced kinase/phosphatase activity, determines pTAU hyperproduction that, in turn, generates pTAU toxic oligomers and pTAU aggregation. Typically, the pathologic pTAU deposition inside the neurons spreads from its initial location in the allocortex to the neocortex. Taken together, oligomeric Aβ, synaptic pTAU aggregates, and glial inflammatory activation represent the main neurotoxic factors underpinning neurocognitive disorders [9,10]. Interpreting the neuropathological picture of AD is a key point in deciphering its pathophysiological mechanisms, from which specific biomarkers and possible therapeutic targets can be identified. Apart from brain atrophy due to synaptic rarefaction and neuronal loss, AD pathology shows an extracellular accumulation of Aβ peptides (Aβ or senile plaques), as well as hyperphosphorylated TAU protein aggregates inside dying neurons called neurofibrillary tangles (NFT) and neuropil threads (NT). Their combination constitutes neuritic plaque (NP), which is the hallmark of AD neuropathology that is scored using the ABC criteria for the *post-mortem* definition of an AD diagnosis. The ABC scoring system includes the following: A as Amyloid-Thal phases for an amyloid; B as Braak stage-a scoring system to grade the pTAU; and C as CERAD (Consortium to Establish a Registry for Alzheimer Disease) score—a scoring system to grade the number of NP [11,12,13].

Senile and neuritic plaques, consisting of protein and cellular debris, initiate reactive and inflammatory cascades involving astrocytes and microglia that produce cytokines (IL-1β and IL-6) and activate NLRP3 inflammasomes that, in turn, increase neurotoxic phenomena [14,15]. Moreover, a recent model for AD pathogenesis involves a modification to the protein expression and compartment functionality in the mitochondria-associated ER membrane. This sub-compartment appears enriched in presenilins and the β-amyloid producing γ-secretase complex in the APP/PS1 mouse model of AD [16]. Mitochondria has also been a focus of interest in AD research due to alterations in the voltage-dependent anion channel 1 (VDAC1) expression and, possibly, functionality [17]. VDAC-1 modulates many mitochondrial functions, including Ca^2+^ homeostasis, oxidative stress, energetic metabolism, and apoptotic cell death. Since overexpression of the anion channel causes apoptosis, and high VDAC1 levels have been found in post-mortem AD brains and in amyloid precursor protein (APP) transgenic mice, it has been hypothesized that VDAC1 may cause neuronal cell death characteristic of AD; therefore, VDAC1 targeting would represent a new way to inhibit neuronal cell death in AD [17].

From the neuropathological picture, specific biomarkers have been obtained, composing the ATN system (Amyloid-TAU-Neurodegeneration). This diagnostic system expresses the AD pathology and allows the in vivo definition of the disease, including the following: 1) A-amyloid evaluation (i.e., a Aβ decrease in the cerebrospinal fluid (CSF) and/or an Aβ cortical accumulation at amyloid-PET); 2) T-pTAU diffusion and topography in the cortex (a pTAU increase in the CSF and/or pTAU cortical accumulation at TAU-PET); and 3) N-neurodegeneration severity (an atrophic pattern in the brain MRI and/or hypometabolism at FDG-PET and/or an increase in total-TAU in the CSF) [18,19]. Biomarkers allow for an early diagnosis and even assist in identifying those most at risk of developing AD while still in the preclinical phase (before mild-NCD), making room for timely therapeutic interventions [20]. Nonetheless, there is currently no cure for AD and this approach poses ethical problems, as well as queries regarding invasiveness and high cost; therefore, a strong effort to identify biomarkers that are obtainable from peripheral blood is still in progress [21,22]. Due to the complexity of AD, the identification of the ideal peripheral markers appears to be a difficult challenge. The early pathogenesis of sporadic AD is quite complex. To exemplify the matter, just as there are different forms of hepatitis that lead to cirrhosis, there are different pathophysiological paths that lead to AD, but the brain is much more complicated than the liver. The early mechanisms leading to an Aβ accumulation and an initial generation of toxic molecules are elusive and multiple, and likely depend on individual trajectories of age-related changes. They depend on both non-modifiable genetic factors (pathogenic mutations in PSN-1-2 and APP genes, APO-E4 allele, AD-related polymorphisms) [23,24], and modifiable factors related to the individual’s history. For effective preventive interventions, modifiable factors are pivotal; they include favorable behaviors (social engagement, healthy diet, high education, regular physical, and mental activity) and detrimental conditions (diabetes, hypertension, midlife obesity, excessive alcohol, smoking, and hearing loss) [25,26]. Education on healthy lifestyles and treatment for midlife diseases are important for people aged 40–50 years old, but this may not be enough if the disease tends to occur anyway. The early pathogenetic role of amyloid burden induced a strength effort to reduce the amyloid load in the brain, especially through the use of costly monoclonal antibodies (e.g., phase three trials: Aducanumab, Gantenerumab; phase two trial: Crenezumab). Actually, lowering the amyloid burden is only one of the possible therapeutic strategies and there is a growing interest in non-amyloid targets, with 121 agents currently being studied in ongoing clinical trials for the treatment of AD [27]. In particular, the development of immunotherapies capable of blocking the toxic oligomers of pTAU represent a promising strategy, and not only for AD [28]. The pathogenesis of AD is multi-dimensional and requires an early and personalized therapeutic approach based on both clinical and biomarker characteristics that are unique and patient specific [29,30,31]. Current therapies for AD can be stratified according to the stage of the disease that identifies the following three levels of intervention: 1) early prevention of risk factors and lifestyle; 2) disease-modifying treatments (a reduction in the load of Aβ and toxic oligomers, the containment of the phosphorylation of TAU and toxic species of pTAU, the control of neuroinflammation, and an improvement of neuronal resilience); and 3) late symptomatic therapies (the modulation of neurotransmitters and an improvement in synaptic efficiency) [32]. However, many senile cases of AD clearly have mixed conditions of brain pathology [33] and, in the extreme stages of senility, it becomes unrealistic to halt neurodegeneration. In this context, purinergic receptors, especially in the hippocampus, constitute a new and interesting target for modulating and improving synaptic activity, and obtaining symptomatic, and possibly disease-modifying, effects.

## 2. Adenosine and AD

**Figure 1:**
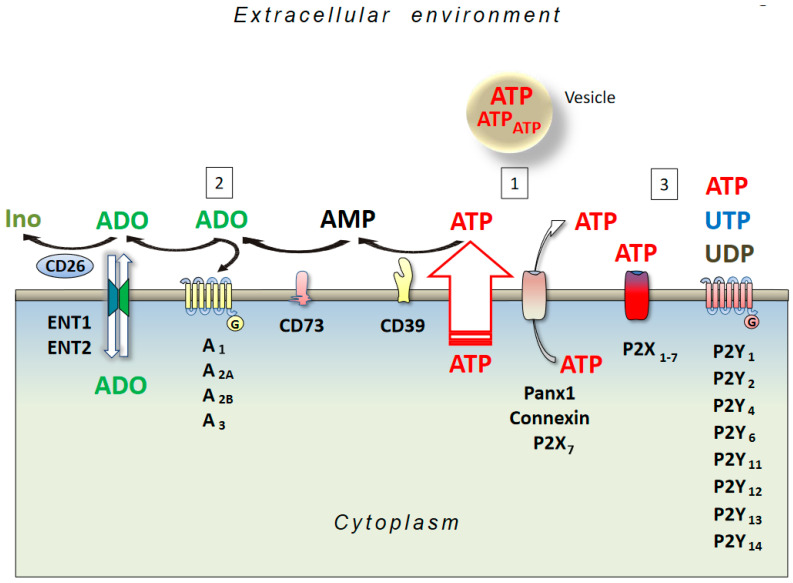
Membrane machinery for nucleotide and nucleoside signaling. (1) ATP can be released by the cells in different ways, among which are membrane stress/damage, and molecular (Panx1, Connexin, P2X7) and vesicular transport. Once on the extracellular side of the membrane, ATP either follows a degradative pathway (2) or stimulates P2 receptors (3). In the first case, ATP, through the activity of the CD39 and CD73 enzymes, is transformed into adenosine (ADO), activating P1 (A_1_, A_2A_, A_2B_, and A_3_) receptors. ADO can also be transported into the cell by ENT1 and ENT2 proteins or be inactivated to inosine (Ino) by CD26.

Adenosine is an endogenous nucleoside, omnipresent throughout the body as it is a degradation product of ATP, whose consumption is ubiquitous. Under physiological conditions, the adenosine concentration inside and outside a cell is 20–300 nM, whilst it increases to micromolar levels following pathologic conditions such as ischemia, hypoxia, and brain injury, again as a consequence of a consistent ATP degradation [34]. Indeed, both an increase in energy consumption or a decrease in energy supply induce extracellular ATP dephosphorylation and adenosine formation due to the activity of ectonucleoside triphosphate diphosphohydrolase (CD39) and ecto-5′-nucleotidase (CD73) enzymes in neurons and glial cells [35]. In addition, at an intracellular level, adenosine may be generated following an intracellular AMP degradation by a cytoplasmic 5′-nucleotidase, or through a hydrolysis of S-adenosyl-homocysteine (SAH) mediated by a SAH hydrolase, and its release is regulated through bi-directional equilibrative nucleoside transporters (ENT) [36] (Figure 1). Then, adenosine may be deaminated or phosphorylated by adenosine deaminase (ADA) or adenosine kinase (AK), with the first pathway being prevalent in pathologic conditions [37].

Adenosine activates four G-protein coupled receptors, A_1_, A_2A_, A_2B_, and A_3_, which are present on a wide range of cellular types, including both neuronal and glial cells [38]. This nucleoside acts as a neuromodulator in the brain, regulating a wide number of physiological effects relevant to AD pathology, spanning from sleep, cognitive abilities, and memory essentially to a control of excitatory synaptic stimulation through inhibitory effects that are mediated by A_1_ receptors and synaptic plasticity induced by A_2A_ receptors [39]. The involvement of A_1_ receptors in AD has been investigated, and it was found that its activation may induce TAU phosphorylation and translocation towards cytoskeleton, possibly avoiding a tangle formation [40]. In addition, its effect on the increase in soluble Aβ has been detected, suggesting a protective role for A_1_ agonists [41]. On the other hand, it has been reported that a fat-enriched diet induced cognitive and memory dysfunctions as well as a reduction in the A_1_ hippocampal receptors that were reverted by the A_1_ receptor’s antagonist, theobromine [42].

As for the A_2B_ and A_3_ receptors, due to their effects on the central nervous system, they may be interesting targets for future investigations. Specifically, it has very recently been found that A_2B_ stimulation reduced the Aβ-dependent cognitive damage in AD-like animals by reducing the Aβ accumulation, cholinergic dysfunction, and mitochondrial toxicity through MAPK activation [43]. Accordingly, a reduction in the A_2B_ receptor expression in selected brain regions was observed in AD, suggesting that A_2B_ could be another therapeutic target of the pathology [44].

The A_3_ receptor in the brain is generally present at low levels in various cerebral regions, comprising of the hippocampus and cortex, and its activation reduced neuropathic pain through N-type Ca^2+^ channel modulation [45,46,47]. In addition, its activation reduced inducible nitric oxide synthase (iNOS) expression, microglia migration, and phagocytosis in BV-2 cells that were stimulated using elevated hydrostatic pressure [48]. Accordingly, contrasting neuroinflammation decreases early brain damage dependent on subarachnoid hemorrhage in elderly animals [49].

A_3_ has also been found to reduce secondary tissue injury and cognitive damage in an animal model of traumatic brain injury [50].

However, among the adenosine receptors, the A_2A_ subtype, which has already been a drug target for Parkinson’s disease (PD) for a long time, is also the one receiving more attention as a therapeutic target in AD [51]. As for its expression, it is particularly abundant in striatal neurons, but it is also present in other areas of the CNS, such as the cortex and hippocampus, where they are located in the synapses and glia [52,53,54]. The effect of its neuronal activation is glutamate release, with consequent NMDA mGluR5-dependent stimulation and a postsynaptic calcium increase, which is responsible for synaptic changes and memory impairment [55,56]. Specifically, the hippocampus is the most important region of the brain for new learning and episodic/spatial memory, where the adult neurogenesis process occurs, limited to the dentate gyrus and the olfactory bulb sub-ventricular zone. Interestingly, through gene analysis studies, *ADORA2A* is significantly associated with hippocampal volume and the presence of its minor allele rs9608282-T, which is responsible for a reduction in its expression, is associated with better memory and a larger hippocampal volume, suggesting that an upsurge of A_2A_ receptor levels in the cortex and hippocampus causes synaptic toxicity and memory deficits [57]. Accordingly, a rapid A_2A_ upregulation within the glutamatergic synapses increases the excitotoxicity through an NMDA receptor recruitment and excessive calcium influx, activating calpains and thus inducing neurodegeneration through mechanisms where glia cells are likely to participate [58].

Indeed, the occurrence of memory impairment in AD is not attributable to a generic neuronal loss, but is linked to the damage of functional synapses, in particular in the hippocampal region at the glutamatergic level [59,60,61]. Adenosine, generated by the ecto-5′-nucleotidase (CD73) enzyme, in animals with early AD, is responsible for the decrease in synaptic markers as wells as for memory damage and LTP disorders through the activation of the A_2A_ adenosine receptor [39,60,61].

Interestingly, the A_2A_ receptor is upregulated in the frontal cortex and hippocampus of elderly animals or in the presence of mutations causing AD as well as in humans affected by AD [39,56,62,63,64,65,66,67,68,69]. The upregulation of neuronal A_2A_ adenosine receptors in APP/PS1 mice impairs long-term synaptic potentiation (LTP) in CA3 pyramidal cells of the hippocampus, affecting their early synaptic deficit [70]. A wide body of literature reports that antagonism of A_2A_ receptors at a synaptic level is responsible for neuroprotection, preventing memory damage in various AD animal models [71]. Specifically, the pharmacological approach of A_2A_ receptor blockade inhibits synaptic injury and cognitive disabilities in animals treated with Aβ, indicating that A_2A_ receptor blockers offer the opportunity to decrease synaptotoxicity and memory impairment [72,73,74]. The same approach restored memory and plasticity in the triple transgenic mouse model of AD, expressing all three pathological hallmarks of AD, characterized by three cumulative mutations in TauP301L, APPSe, and γ-secretase (PS1M146V), that mimicked several features of AD, including the early memory deficits [61]. Similarly, the antagonism of A_2A_ adenosine receptors using both gene silencing and pharmacological block in THY-TAU22 mice, ameliorates the TAU pathological phenotype by decreasing TAU hyperphosphorylation and aggregation, inflammation in the hippocampal region and, at the same time, saving spatial memory and hippocampal long-term depression [75]. The involvement of A_2A_ receptor upregulation in tauopathy, TAU hyperphosphorylation, and memory damage was confirmed in a more recent study [76].

Several studies have revealed a role of the A_2A_ receptor as a druggable protein in AD, not only in neurons but also in microglia, to restore memory impairment and counteract neurodegenerative processes [71,72,73,74,75,76,77]. In this context, it is known that the essential role of the A_2A_ adenosine receptor is the regulation of glial cell functions, addressing cytokine release, and thus inducing neuroinflammation [78]. Among glial cells, microglia are particularly important due to the increase in A_2A_ receptor expression in activated cells that are located near the amyloid plaques that are typical of AD. Overexpression of this adenosine receptor subtype induces an increase in cytokines, including IL-1β, IL-6, and TNF-α, while its blockade hampers hippocampal LTP disorders and IL-1β secretion, playing a key role to decrease the memory deficit [79,80]. The upregulation of A_2A_ receptors that occur in microglia also expressing NMDA receptors is also of interest [81]. Specifically, it has been shown that they may form, both in neurons and microglia, A_2A_-NMDA heteromers, interacting in a reciprocal way, where A_2A_ receptor blockers antagonize an NMDA-dependent calcium increase [56,81,82,83]. Of particular relevance in microglia is the presence of A_2A_-CB_2_ heteromers, upregulated in AD, where A_2A_ receptor antagonists are responsible for the increase in CB_2_ receptor activity, thus potentiating the neuroprotection mediated by endocannabinoids [79,80,81] (Table 1).

The clinical relevance of A_2A_ adenosine antagonists to avoid memory impairment is attested by human studies demonstrating the efficacy of caffeine intake in the prevention of cognitive dysfunction in the elderly [103,104,105,106,107,108,109,110,111]. Indeed caffeine, the most widely consumed psychostimulant substance, present in coffee, tea, cola, chocolate, and other foods, behaves essentially as a blocker of A_2A_ receptors in the brain [112,113,114]. Accordingly, a 20-year retrospective study devoted to revealing whether caffeine intake could protect against AD demonstrated an inverse correlation between coffee consumption and disease development [115]. In addition, a five-year prospective analysis of the Canadian population investigating the consequence of daily coffee consumption on AD progression indicated an odds ratio of 0.69 that is suggestive of a significant reduction in AD risk [116]. In particular, an intake of three cups of coffee per day was inversely related to a decrease in cognitive function [117,118]. Accordingly, an intake of 3–5 cups of coffee/day decreased the risk of dementia and AD by 65–70% and 62–64%, respectively, versus a minor consumption [103]. Further studies reported that patients affected by MCI, who later progressed to dementia, presented lower plasma levels of caffeine in comparison to stable MCI patients during a period of 2–4 years [119]. Interestingly, it has been suggested that caffeinated coffee consumption was associated with this protection probably hampering a specific immune deficit linked to a decrease in G-CSF, IL-10, and IL-6 levels in MCI patients several years prior to dementia conversion [119]. In particular, as for G-CSF, it affects synaptogenesis, neurogenesis, and Aβ cerebral phagocytosis by immune cells, thus providing cognitive improvement in AD mice [119]. Accordingly, a recent clinical trial with sargramostim, a recombinant GM-CSF, found an increase in activated microglia, a 50% decrease in amyloid content, a rise in the synaptic area, and an amelioration in spatial memory [120]. Indeed, its blood levels, together with that of other neurotrophic/hematopoietic factors (e.g., BDNF, SCF), have been found to be reduced in early AD [121], resulting in deficient neurotrophic/hematopoietic brain support. The role of the immune system and inflammation is quite complex; when microglial cells are moderately activated and specifically induced to remove an amyloid (immunomodulatory, monoclonal-Ab, and active immunization treatments), the effect can be favorable. Unfortunately, in the elderly, immunological senescence and “inflammaging” chronic phenomena lead to an excessive inflammatory activation and a reduction in the specific adaptive responses of the immune system. Therefore, the loss of balance in the processes of immunity and inflammation, especially in old age, results in damage [84,85,122,123].

Further studies confirmed the beneficial effects of caffeine consumption in humans, with the lack of dementia and cerebral damage typical of AD, and to a rise in long-term memory consolidation [105,124,125]. Furthermore, the administration of caffeine to animals with AD revealed a lower risk of memory disabilities, and less beta-amyloid accumulation and TAU hyperphosphorylation [104,108,126,127,128,129,130,131,132], whilst in SH-SY5Y cells antioxidant and anti-inflammatory properties have been detected [133,134]. However, it has to be underlined that coffee contains caffeine as well as more than 2000 other substances that may have biological activities. At this proposal, it has been reported that the CSF levels of theobromine, a metabolite of caffeine, in demented patients or its intake through chocolate is inversely correlated with memory disabilities [135,136].

The neuroprotection exerted by caffeine is not a novelty, as there is a huge amount of literature reporting that an A_2A_ adenosine receptor block is also beneficial in the case of PD, where istradefylline, an A_2A_ adenosine receptor antagonist, has been launched as a new drug for the treatment of this disease in Japan (Nouriast) and in the US (Nourianz) due to its safety and efficacy [38,137,138]. On these bases, it would be important that future studies also establish its efficacy in the reduction in memory impairment in patients with AD [74]. This evidence would allow for a rapid entry of this drug on the market for AD treatment.

## 3. ATP and AD

Membrane receptors for the extracellular nucleotides are widely distributed in eukaryotic cells. They are activated by nucleotides such as ATP, ADP, UTP, UDP, etc., which are released from the cells or synthesized extracellularly, where they bind to ionotropic P2X and G-protein-coupled P2Y receptors. Seven P2X receptor genes have been identified in humans, while there are eight for P2Y receptors. P2X subunits can assemble as homo- or hetero-oligomers, and ATP is an agonist for all of the P2X subtypes.

P2Y receptors are seven domain membrane-spanning molecules that bind to G-proteins and show different ligand affinities for uridine and adenine nucleotides. According to their gene sequence, G-protein coupling, and agonist affinity, they are divided into two subgroups. One includes the P2Y_1_, P2Y_2_, P2Y_4_, P2Y_6_, and P2Y_11_ receptors, while the other comprises of the P2Y_12_, P2Y_13_, and P2Y_14_ subtypes. Gq/G11 proteins couple to the first receptors, whose activation generates inositol-1,4,5-triphosphate and evokes a Ca^2+^ liberation from the endoplasmic reticulum. The latter receptors couple to Gi/G0 proteins, causing adenylyl cyclase inhibition [139]. A plethora of scientific reports have documented the multiple effects of P2 receptors in human tissues [140]. Concerning the central nervous system (CNS), neuronal cells express P2X and P2Y receptors particularly of the following subtypes: P2X2, P2X4, P2X4/P2X6, and P2Y_1_ [141]. In neuronal cells, P2 localization can be pre- or postsynaptic. Differences exist between pre-synaptic expression of the P2X and P2Y receptors, that may be either excitatory (P2X) or inhibitory (P2Y), while post-synaptic P2 subtypes are always excitatory [141]. In the CNS, microglial cells also express different P2 subtypes (P2X4, P2X7, P2Y_6_, and P2Y_12_) that are able to profoundly modulate responses, particularly those that are linked to inflammation [88,89,90]. Astrocytes also express P2 receptors that are involved in their activation and in the release of cell factors promoting neuronal damage repair and axonal regeneration [142]. Different P2X subtypes have been linked to pain perception, among them, the neuronal P2X3, P2X2/P2X3 heterotrimers, and microglial P2X4 receptor [143].

The P2X7 subtype has attracted a lot of interest among immunologists as it is widely expressed in cells of the innate immune system and has an ability to modulate the immune response. Among the cells expressing high levels of this subtype are macrophages, microglia, dendritic cells, but also B and T lymphocytes. Moreover, it is present in different regions of the CNS, i.e., the frontal cortex, hippocampus, amygdala, and striatum, making it an interesting molecule to bridge neuroimmune response and brain dysfunction [144]. Hence, P2X7 activation has been implicated in pathological neuroinflammation, neuronal damage, and death [145]. Moreover, a number of influential studies have linked P2X7 to neuropsychiatric disorders and diseases. In particular, receptor single nucleotide polymorphisms have been associated with anxiety, bipolar disorder, depression, multiple sclerosis, Parkinson’s, and Alzheimer’s disease [144,146].

The formation of Aβ plaques and neuronal cell deaths are hallmarks of AD [147] and, as shown in the mouse model, both Aβ and its precursor are potent neuroinflammatory stimuli heavily compromising blood circulation in the brain. However, quite a recent and important acquisition in understanding the pathogenesis of AD has consisted of the identification of co-inflammatory agents that are able to increase the deleterious effects of Aβ. Extracellular ATP can burst inflammation and cause neuronal damage by activating the P2 receptors expressed by microglia, astrocytes, and neuronal cells [148]. From a mechanistic point of view, it can be hypothesized that the activation of the P2X receptors may cause neuronal stress and death due to excessive Ca^2+^ entry increasing the cytoplasmic Ca^2+^ concentration and causing mitochondrial Ca^2+^ overload, up to levels that undermine the activity of the organelles, with a formation of radical species and the release of pro-apoptotic factors. An accumulation of Aβ also increases the intracellular Ca^2+^ in neurons, both by causing an external Ca^2+^ influx and a release of the ion from the intracellular stores. Excessive long-term depression causing progressive memory decline and increased neuronal apoptosis in AD patients has been hypothesized to depend on an upregulation of Ca^2+^ signaling [149]. Moreover, the Aβ_25–35_ fragment evokes intracellular Ca^2+^ concentration changes via connexin hemichannel opening and purinergic receptor activation in astrocytes, whose exposure to Aβ_25–35_ induces both a Ca^2+^-independent and Ca^2+^-dependent glutamate release in the brain of the hAPPJ20 AD mouse model [150].

Microglia were found to be particularly important for the setting of the inflammatory background underlying AD as Aβ induces an ATP release from these cells and activates them to migrate towards the plaques. Moreover, ATP-stimulated microglia release inflammatory cytokines and toxic mediators, contributing to the neuronal cell death that is evident in AD [91,92,93].

Different P2 receptors play a role in inflammation. Among them, it is long known the fundamental role of the P2X7 subtype in bursting and sustaining both sterile and non-sterile inflammation [94]. Since AD has been interpreted as a pathological modification of the central nervous system due to a chronic inflammatory status, investigations have increasingly concentrated on P2X7. Expression of the receptor was found increased in an AD mouse model, the P301C TAU transgenic mice [151], as well as upon the intrahippocampal injection of Aβ in Tg2576 transgenic mice and rats [152]. Similarly, P2X7 upregulation is present in the brain tissue of AD patients, and molecular modifications of the subtype have been also described, although their significance has not yet been interpreted [153,154]. Another peculiarity of the P2X7 receptor expressed in AD patients is the presence of two SNPs, i.e., the 1513A > C (rs3751143) and 489C > T (rs208294). The contemporary presence of these modifications would contribute to the disease pathogenesis [155].

The functional involvement of P2X7 in AD has been intensively investigated and different responses mediated by the receptor have been linked to the tissue modifications found in AD animal models and in humans. The involvement of P2X7 in the secretion and cleavage of pro-IL-1β in macrophages and microglia have been widely documented in different systems and with various experimental setups [156]. Accordingly, the intracellular pro-IL-1β of the P2X7^−/−^ mouse macrophages is not activated by caspase-1 and externalized [157]. Moreover, general in vivo inflammatory response and leukocyte activity is less pronounced in animals lacking the receptor [95]. It is of particular interest for AD pathogenesis, as the activation of microglia by Aβ requires P2X7 expression [93]. Accordingly, P2X7 pharmacologic inhibition obliterates Aβ-induced microglial activation [96]. Mechanistically, Aβ evokes the liberation of intracellular ATP that, in turn, causes the release of the pro-inflammatory cytokines IL-1β, TNF-α, IL-18, and IL-6 in rat and mouse microglia [86,87,97]. Interestingly, these cytokines have been indicated as crucial molecules in AD and their concentration is increased in AD patients’ brains [98,99,100]. Another recently identified P2X7-related pathway leading to pathogenic consequences is that involving CCL3 chemokine secretion in an AD mouse model. In this study, the authors showed that Aβ stimulated a CD8^+^ lymphocyte recruitment [101]. Moreover, an Aβ peptide increases P2X7 expression in microglia and a rat’s hippocampus, and activation of the subtype causes neuronal apoptosis [158,159]. P2X7 receptor activation has been also linked to the secretion of APP fragments in different experimental models. In particular, the receptor caused the formation of non-amyloidogenic APP by metalloproteases [160]. The process was totally dependent on P2X7 receptor expression and was absent in P2X7-/-cells. Moreover, the fact that sAPPα, and not Aβ, fragments were found upon activation of P2X7 has prompted researchers to hypothesize that the α-secretase pathway was mainly stimulated by the receptor. However, different studies have produced contrasting results, i.e., the prevention of α-secretase activity by P2X7 activation [161,162,163] (Table 1).

Less information is available on the involvement of the P2X2 subtype in AD. A pretreatment of PC12 cells and hippocampal neurons with an Aβ-increased P2X2 expression and ATP induced a Ca^2+^ increase [164]. P2X4 stimulation would augment Aβ_1–42_-mediated toxic effects, making it also a potential pharmacologically targetable molecule for AD [165,166].

P2Y receptors have also been linked to AD, although their signaling and role have not been completely characterized in AD tissues. So far, the majority of P2Y-mediated responses appear to be neuroprotective and even able to prevent Aβ deleterious effects, with the exception of the P2Y_1_ subtype [161,167,168]. This subtype is highly expressed in the reactive astrocytes surrounding plaques and was found responsible for causing astrocytic hyperactivity in APPPS1 mice, a mouse model of AD. Accordingly, inhibition of the P2Y_1_ receptor normalized the astroglial network dysfunction [169].

P2Y_1_, P2Y_2_, P2Y_4_, P2Y_6_, and P2Y_12_ receptors seem more involved in brain changes found in AD. P2Y_1_ is involved in astrocyte hyperreactivity and its inhibition reduces neuronal damage and synaptic dysfunction, thus preserving spatial learning and memory, in mice [168]. The P2Y_2_ subtype stimulates the degradation and uptake of Aβ_1–42_ [170], with P2Y_4_ playing a similar role [171], while P2Y_6_ and P2Y_12_ would be involved in modulating the removal of dead neurons by the microglia [165,166,167,168,169,170,171,172].

## 4. Conclusions

Different approaches have been tested to treat AD. Trials using pharmacological drugs that aimed to lower levels of Aβ and prevent its deleterious accumulation in the brain have unfortunately been unsatisfactory; therefore, alternative strategies have to be taken into account.

Coffee, the most consumed beverage in the world, contains caffeine, a famous psychoactive substance that, following regular consumption, is associated with a reduction in AD insurgence in humans. Its mechanism of action is linked to an interaction with the A_2A_ adenosine receptor, whose level of expression and transduction machinery is deeply modified in the aged hippocampus, where it undergoes overactivation, leading to an increase in glutamate synapsis functioning, with mGluR5-dependent NMDA receptor overstimulation, leading to a calcium overload that is a typical hallmark of aging. Several works agree to indicate, either by antagonizing A_2A_ or silencing it, that its removal is responsible for beneficial effects in both aged and animal AD models [173]. Even though further work is necessary to clarify all the aspects related to the effects of A_2A_ receptor antagonism in AD, the premise regarding its capability to affect synaptotoxicity, glutamate transmission, calcium overload, as well as neuroinflammation, are encouraging and suggest a key role for its antagonism as a novel approach to avoid AD insurgence.

Another likely practicable approach may be to counteract neuroinflammation, whose signs (microglial activation and the presence of inflammatory cytokines) have been found in post-mortem samples of AD patients’ brains [172]. Since the P2X7 receptor plays a pivotal role in inflammation, different clinical trials have been undertaken in humans with specific inhibitors of this subtype. They are all aimed at treating diseases where chronic inflammation plays a prominent role [174]. Antagonists of the P2X7 receptors are obviously also good candidates to counteract the dangerous effects of chronic brain inflammation and pathological responses arising in the central nervous system [175]. The intense research on these molecules has contributed to the development of selective and effective pharmacological inhibitors. Some of them are also endowed with the ability to pass the hematoencephalic barrier, thus permeating the cerebral tissue [102,176,177].

Inhibition of the P2X7 receptor to block its proinflammatory effects on Aβ has been questioned by some authors that found spatial memory impairment and the alteration of other behavioral parameters in P2X7^−/−^ mice and in animals in which P2X7 was pharmacologically inhibited. It has to be said that these results were obtained in AD animal models and, so far, no one can predict the consequences of P2X7 antagonization in the human brain [102,178,179].

Brilliant blue G (BBG), which has been widely used in in vitro experiments as an effective P2X7 blocker, crosses the blood–brain barrier, therefore, it was also employed in vivo animal experiments with positive results [180]. Among recently developed P2X7 targeting molecules, we can mention compounds that are going to be tested in different animal models, i.e., CE-224535 by Pfizer, GSK1370319A and GSK1482160 by GlaxoSmithKline, and JNJ-54175446 and JNJ-55308942 by Janssen laboratories [175,181,182,183,184,185,186,187]. The P2Y_1_ subtype seems to be a good candidate to normalize the astrocyte hyperactivity, glio-vascular signaling, and overall neuronal functionality as in vivo experiments with blockers of the receptors showed very positive results in a mouse model of AD [169].

Another strategy involving nucleotides and their pharmacological analogues as potential pharmacological molecules in AD has taken into account the oxidative stress characteristic of the disease [174,175]. ATP-γ-S, the non-hydrolysable ATP analog, is a recognized P2 receptor agonist but is also an antioxidant with neuroprotective properties. The chemical modification of this molecule has further increased its antioxidant capacity and stability, making it very interesting to try to reduce neuronal mortality [188,189,190,191]. Hence, both adenosine- and ATP-mediated purinergic signaling seem to be promising ways to treat AD.

## Figures and Tables

**Table 1:** Purinergic mediated responses and role in AD of P1 and P2 receptors expressed in neuronal, astrocytic, and microglial cells.

Purinergic Receptor	Cell Type	Response	Role in AD	Refrerences
A1	Neuron	TAU translocation	positive	[36]
		Non-Am. APP Processing	Positive	[36]
A_2A_	Neuron	Aβ-Induced Neurotoxiciy	Negative	[54,59,60,66,68,69]
	Neuron	Increased Aβ accumulation	Negative	[84,85]
	Neuron	Tau pathology	Negative	[71,72]
	Neuron	Memory damage	Negative	[84]
	Astrocytes	Memory function	Negative	[62]
	Microglia	Neuroinflammation	Negative	[75,76]
A_2B_	Neuron	Mitochondrial function	Positive	[39,40]
A_3_	Microglia	Decrease of inflammation	Positive	[44]
P2X4	Neuron	Neurotoxicity	Negative	[86,87]
P2X7	Microglia	Inflammation	Negative	[88,89,90,91,92,93,94]
	Neuron	Amyloidogenic	Negative	[95,96,97]
P2Y_1_	Astrocyte	Hyper-Activation	Negative	[98]
	Neuron	Synaptic damage	Negative	[98]
P2Y_2_	Neuron	Neurite Development	Positive	[95]
	Neuron	Non-Am. APP Processing	Positive	[99]
P2Y_4_	Microglia	Uptake Aβ_1-42_	Positive	[100]
P2Y_6_	Microglia	Phagocytosis	Positive	[86]
P2Y_12_	Microglia	Migration	Positive	[101]
P2Y_13_	Neuron	Decrease ROS-induced death	Positive	[102]
